# A Novel Approach to Monitoring the Curing of Epoxy in Closed Tools by Use of Ultrasonic Spectroscopy

**DOI:** 10.3390/s18010096

**Published:** 2017-12-31

**Authors:** Christian Pommer, Michael Sinapius

**Affiliations:** Institut for Adaptronics and Function Integration, Technische Universität Braunschweig, Langer Kamp 6, 38106 Braunschweig, Germany; m.sinapius@tu-bs.de

**Keywords:** ultrasonic, cure monitoring, resonant ultrasonic spectroscopy

## Abstract

The increasing use of composite materials has led to a greater demand for efficient curing cycles to reduce costs and speed up production cycles in manufacturing. One method to achieve this goal is in-line cure monitoring to determine the exact curing time. This article proposes a novel method through which to monitor the curing process inside closed tools by employing ultrasonic spectroscopy. A simple experiment is used to demonstrate the change in the ultrasonic spectrum during the cure cycle of an epoxy. The results clearly reveal a direct correlation between the amplitude and state of cure. The glass transition point is indicated by a global minimum of the reflected amplitude.

## 1. Introduction

Composite materials offer high stiffness at low weight. That makes them an ideal material for aeronautical and space applications and even for lightweight automotive parts. This broader range of applications has led to the need for large quantities of composite materials at lower prices and with consistent quality. This in turn provides new challenges in manufacturing. In particular, the curing time is a challenge, as faster production cycles are needed.

Currently, most manufacturers use standard curing cycles with high safety factors to determine the cure duration. Long curing times lower the productivity and can imply a risk of products not fully being cured. A possible solution to the issue is the application of cure monitoring. This allows optimization of curing cycles, resulting in higher productivity.

Different cure monitoring techniques are available, like dielectric cure monitoring [1,2], which is already well established, and fiber optic cure monitoring [3,4]. Just a few cure monitoring techniques are able to detect cure changes without direct contact with the composite material. Direct contact may result in preventable wear of sensors and tooling. Moreover, the creation of undesirable visible production marks is most likely. Ultrasonic cure monitoring is one of the few techniques able to observe curing without involving direct contact [5], as it detects the change in acoustic impedance and damping of the resin during the process. This paper focuses on a different approach to ultrasonic cure monitoring. This method actively uses acoustic tool resonances with low energy and low cost.

## 2. Resonant Ultrasonic Spectroscopy for Cure Monitoring

While pulse-based ultrasound cure monitoring is already well established [5], it requires high-speed and highly-sensitive digital processing. These characteristics are usually mutually exclusive, and a compromise is required between speed and resolution. An alternative to pulse-based ultrasonics is the active utilization of tool resonances to create high-amplitude and highly-sensitive measurement signals.

This principle utilizes the acoustic border absorption between tool and material to determine the state of cure. The absorption of acoustic waves at the border is determined by the density ρ and the speed of sound *c* of the tool “tool” and the material “mat” as illustrated in Equation (1).

(1) $$\begin{array}{cc} r & =\left(\frac{Z_{mat}-Z_{tool}}{Z_{tool}+Z_{mat}}\right)\;\;\;\left(Z_{tool}=const.\right)\\ r & \propto \left(\rho_{mat}\cdot c_{mat}\right) \end{array}$$

While this equation does not require the use of a resonance, the active utilization of such resonances can reduce the overall power consumption by producing sufficiently high output signals with low power actuation.

**Figure 1:**
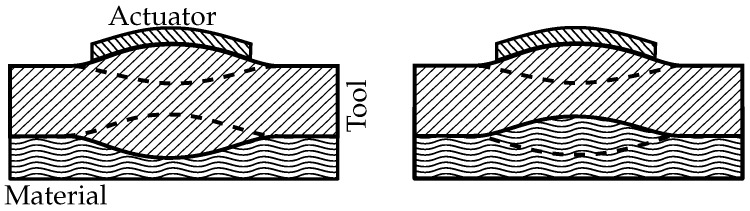
Different resonance types below the actuator. (symmetric, left; anti-symmetric, right).

A typical tool used for composite production processes has an infinite number of resonance frequencies and eigenmodes. The location and size of those eigenmodes is a crucial factor for the localization and accuracy of this method. The distance between actuator and eigenmode should be as short as possible to minimize the area where energy can be lost. A good way to achieve this is the utilization of the resonance of the shortest way between actuator and material as illustrated in Figure 1 for a plate type tool.

The eigenmodes displayed produce symmetric and anti-symmetric guided waves. To measure the amplitude of the resonances, a separate sensor is highly beneficial. The sensor detects the guided waves or other acoustic emissions by the eigenmode. The amplitude of this eigenmode is directly influenced by the state of cure as the epoxy changes density and creates an additional damping effect, not described in Equation (1), as a result of internal viscosity changes.

## 3. Published Knowledge

To determine the current state-of-the-art of the ultrasound resonant spectroscopy technique for cure monitoring, an extensive literature review was conducted. Table 1 and Table 2 show the results for a maximum of 500 relevant articles.

The survey reveals that for the proposed technique, there is currently just a small research base, with a total of two relevant articles found compared to the 32 relevant articles for pulsed ultrasonic cure monitoring. Although the keywords indicate their possible relevance, 465 articles do not contribute to cure monitoring or are inaccessible.

The article *“Cure monitoring of thin adhesive layers”* [6] uses a spectral approach to determine the cure of thin adhesive layers between two metal rods. The author uses a transmission approach to determine the shear wave transmission between two rods to determine the state of cure. When cross-linking during cure occurs, shear waves get transmitted, resulting in a clearly visible additional frequency. The approach requires an acoustic transmission, which is challenging. Furthermore, only the cure of thin layers is observed.

The paper *“Cure monitoring of carbon epoxy composites: an application of resonant ultrasound spectroscopy”* [6] proposes a similar approach, but with a vastly different technique. The paper reports the experimental result of a cure monitoring experiment of a vacuum-sealed bagged prepreg on a coil plate. The author uses a high-power ultrasonic horn to drive the ultrasonic waves.

The results reveal a direct influence of the ultrasonic response on the cure of the prepreg material. The approach itself focuses on the evolution of the frequency shift. The shift is rather small and is heavily influenced by the exothermal reactions of the epoxy. Thus, the method does not provide satisfying results. Furthermore, the use of a high-power ultrasonic horn may severely influence the frequencies. It consists of a high thermal mass, which, in turn, can create unwanted residual stress. Moreover, the method is not cost-efficient since it requires high power and highly specialized equipment. In addition, the frequencies proposed are resonance frequencies of the whole plate, which does not allow localized cure monitoring. The technique that is proposed in Section 2 aims at a local eigenmode, in contrast to the global eigenmode proposed in [6]. This allows for better localization.

A further detailed search for additional literature based on [6] yielded six additional citations. Surprisingly, they did not contribute to curing or cure monitoring.

## 4. Materials and Methods

A simple experiment was able to investigate the feasibility of ultrasonic spectroscopy to monitor the curing of epoxy materials. An epoxy (RTM6 from Hexcel) was applied to an aluminum plate. This epoxy represents many matrix systems with similar properties. The plate itself had dimensions of 250 × 250 × 20 mm. The dimensions were chosen to provide a satisfying target area at a manageable size while maintaining a plate.

Two piezo-ceramic converters made of PIC255 were applied on the back side close to the center with a 40-mm distance between each other. One converter was operated as a transmitter, and the other one as a sensor. The epoxy used to fix the sensor and actuator was also RTM6. It had been previously cured at 180 ${}^{\circ }$C under a vacuum.

**Figure 2:**
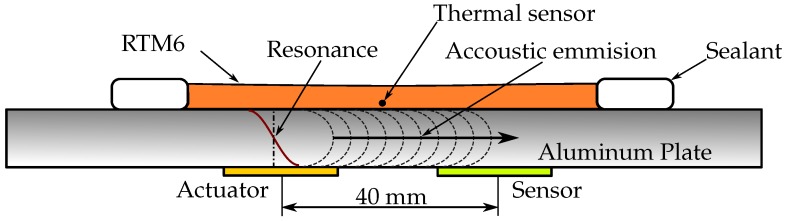
Test setup and principle for ultrasonic spectroscopy cure monitoring of the epoxy RTM 6with a piezoelectric actuator and a piezoelectric sensor.

On the front side, a basin was formed using a general vacuum sealant. A standard thermal sensor was applied in the center of the basin. First, the plate was heated to 80 ${}^{\circ }$C. Then, the cold (25 ${}^{\circ }$C) epoxy was filled in the basin, and the setup was placed in an oven at 180 ${}^{\circ }$C, where it was left until it was fully cured. This setup is displayed in Figure 2.

During the whole time, the ultrasonic spectrum was measured using a 65- and 90-kHz discrete 300-point swept sine actuation with a 20-V peak-to-peak amplitude. This frequency range is in direct proximity to the first resonance frequency in the plate thickness direction and has sufficiently strong resonant amplitudes.

The resonance frequency range is estimated:

(2) $$f_{n}=n\cdot \frac{c}{2d}\;\left(n=1,2,3,\ldots \right),$$

where $f_{n}$ is the resonance frequency, *c* is the speed of sound, *d* is the thickness of the plate and *n* determines the resonance order. By using the known sound parameter for aluminum and the thickness of 20 mm, the fundamental resonance frequency is calculated at 77 kHz for shear waves.

## 5. Results

**Figure 3:**
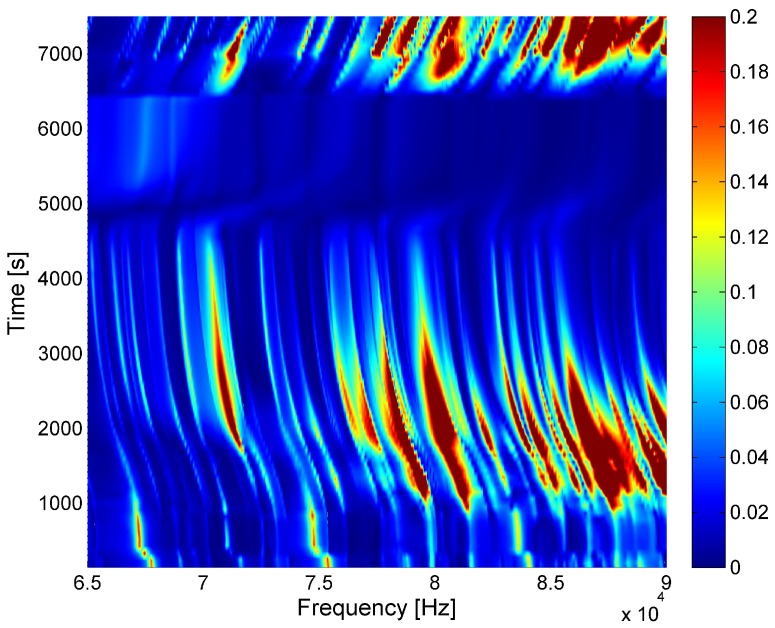
Change in the response spectrum during curing over time.

Figure 3 shows the results of a single cure measurement using ultrasonic spectroscopy in a range of 65–90 kHz during the cure of RTM6. The abscissa shows the frequency (Hz); the ordinate axis the time (s); and the color represents the amplitude (V) of the harmonic response signal.

Although only the first resonance frequency in the plate thickness direction is targeted, multiple resonance frequencies are excited. They are a result of inaccuracies in the plate manufacturing, as well as interferences by reflections at the boarders of the plate.

Figure 3 shows a number of interesting points in time. The first one can be seen at the very bottom. A significant and very fast shift of most resonance frequencies can be seen at approximately 400 s. At this point, the epoxy is filled into the basin. The fact that not all resonances are affected in the same way is a clear indicator of the different resonance types. The resonance spectrum itself is a result of all resonances of the sensor-actuator-plate system. This implies that it contains the resonances of the actuator and the sensor, as well. Their frequencies are not affected by the epoxy on the other side of the plate. Nonetheless, their amplitude is affected by the amplitude of the reflected waves as well.

The second interesting point is the frequency shift, which starts at approximately 1000 s. This is the result of a rise in temperature in the pre-heated oven. It affects all frequencies in the same way. The amplitudes are particularly interesting. Most resonance amplitudes are growing at the beginning, while they decrease towards the end. This is a result of the change in epoxy parameters, as well as sensor-glue parameters. A temperature-induced reduction of the glue stiffness results in a reduction of the resonance of the actuator/glue system, bringing it closer to the measured frequency band. This leads to an increase in sensitivity, as well as the actuator output. While the impedance of the aluminum does not change much during heating, the acoustic impedance of the epoxy is decreasing as a result of the drop in density. This explains the high amplitude change during the heating process, but not the diminishing resonance signals as time goes on. The diminishing is a result of a different effect, which cannot be explained by temperature changes. It is a direct effect of the slowly progressing cure process. While the temperature rise results in a thermal expansion, the curing progressively shrinks the epoxy, adding an increasing stiffness, as well as significant damping because of the increasing viscosity.

This directly leads to the third interesting point in time, at approximately 5000 s, where the glass transition occurs. The epoxy has nearly no fluid properties any more. The glass transition point can be accurately pinpointed by measuring the viscosity or approximated by calculating the degree of cure as done later. The viscosity, on the other hand, directly affects the damping of the material. As the fluid parameters decrease, the damping is increasing, reaching an absolute maximum in the glass transition point. This effect affects all sound waves hitting the boundary line between the epoxy and the metal, diminishing them to a global minimum. As is clearly visible, all resonance amplitudes are affected in the same way. The frequency shift is a result of the change in boundary conditions.

After the glass transition, the inner viscosity of the newly-formed solid body falls to its final, far lower, value. This change can be seen in the resonance spectrum, as well where the resonance amplitudes are returning and a new weaker resonance spectrum becomes visible. This is a clear indication of the “after cure” region.

The fifth change in the impedance spectrum occurs when the oven is opened at approximately 6500 s and the system is rapidly cooled. Due to the difference in the specific thermal expansion parameters, a high shearing force between the metal and the epoxy occurs, leading to complete detachment. This is clearly indicated by the step in amplitude and can be visibly confirmed during the experiment.

**Figure 4:**
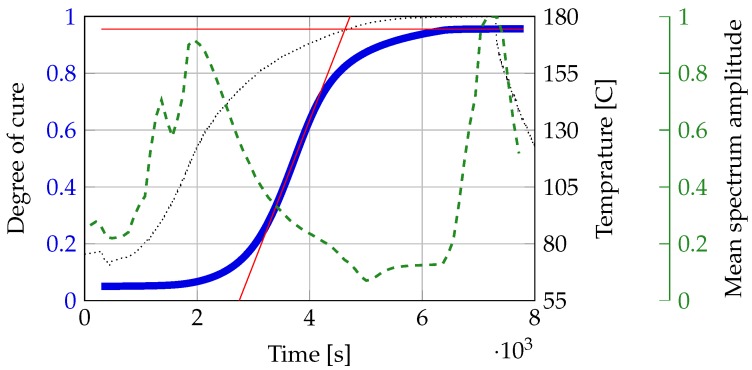
Measured temperature (black-dotted line) and calculated degree of cure (blue line) of the epoxy during the experiment with added support lines (in red) to determine the glass transition point (intersection). The mean spectrum amplitude of Figure 3 is shown in the green-dashed line.

Figure 4 shows the measured temperature change, the calculated curing of the epoxy and the change of the mean value of the amplitude of Figure 3. The calculations are based on the dissertation of Karkanas [7]. The figure clearly shows a similar point of cure at the measured temperature profile. The bump in the temperature profile at around 300 s is a result of the epoxy being poured in. The epoxy was kept at room temperature. The results clearly show that the minimum of the mean spectrum is very close to the calculated point of cure. Differences can be a result of calculation and epoxy variations.

**Figure 5:**
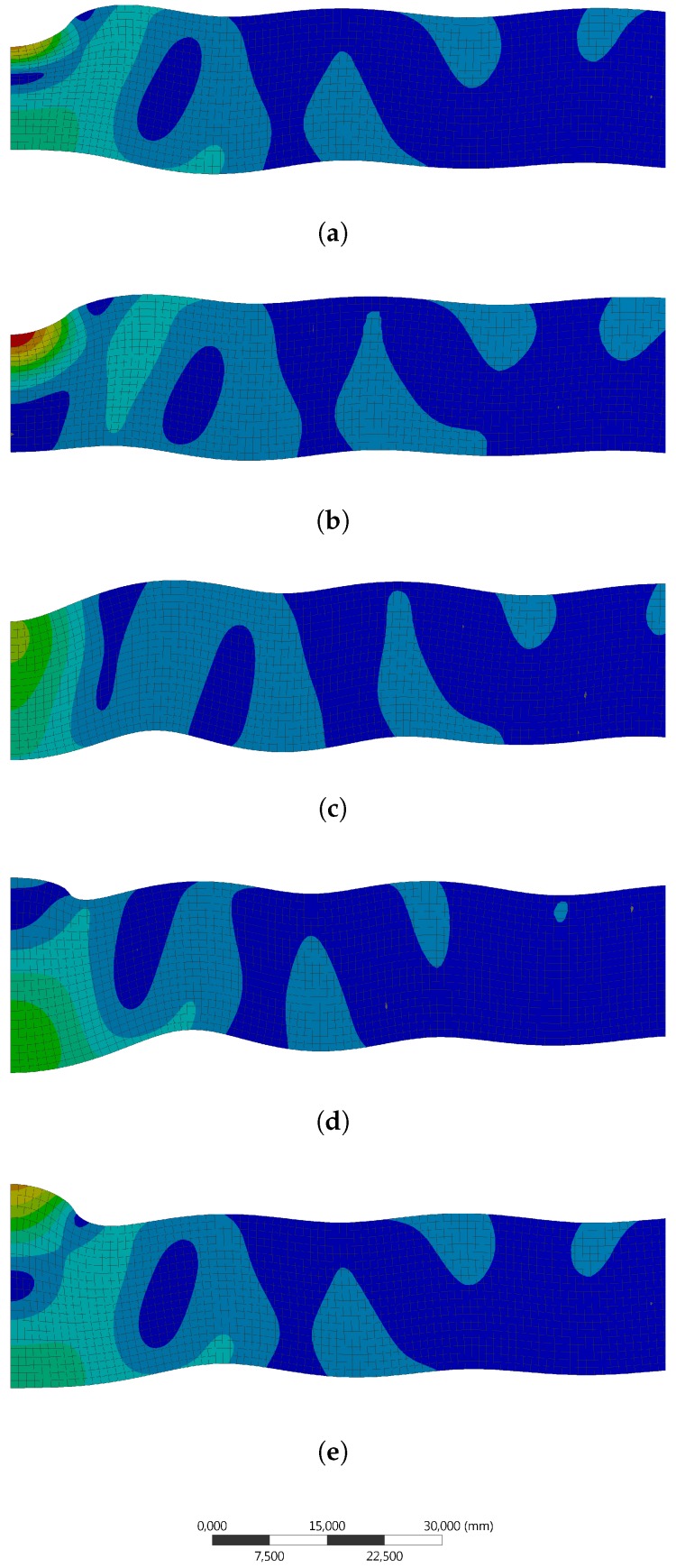
FEM vibration analysis of 80,300-Hz amplified excitation at different phase angles with a 1-mm ring element-size; center left-plate continues to the right. (**a**) 0${}^{\circ }$; (**b**) 45${}^{\circ }$; (**c**) 90${}^{\circ }$; (**d**) 135${}^{\circ }$; (**e**) 180${}^{\circ }$.

A 2D FEM-simulation is used to simulate the vibration of the aluminum plate at 80 kHz by using an endless plate approach and ring elements with a 1-mm edge size. The plate is actuated by a pressure from the top with a radius of 8 mm. This is the same radius as the actuator used in the experiment. The FEM-simulation displays a combined actuation of the two eigenmodes as shown in Figure 1. Figure 5 illustrates the deformation at different phase angles. The simulation however does not take the epoxy into account, which might have a considerable influence.

## 6. Discussion

The results presented in Section 5 show a very clear correlation between amplitude and point of cure visible in Figure 3 and Figure 4. There is only a small deviation between calculation and measurement. A possible reason for this deviation is an erroneous temperature measurement. The temperature sensor is very close to the aluminum plate. This creates a faster temperature rise. A fitting error in the curing lines might be a source of deviation, as well. The deviation is however quite small and can be disregarded.

The results of the experiment clearly show the effectiveness of the presented method. Measuring the resonance spectrum allows for good detection of the glass transition point. Figure 3 also shows that the exact frequency is not important. All frequencies are diminished equally. This is a result of Equation (1). This allows for tracking of epoxy cure even in closed tools without direct contact between the epoxy and sensor. The removal of a direct sensor epoxy contact, which is always accompanied by high abrasion before, after or during the cure, possibly allows for use in far rougher environments, which are quite common in industrial manufacturing processes.

As only the amplitude is important, the manner of analysis can be changed. While pulsed ultrasound uses most often digital analysis, resonant ultrasonic spectroscopy just requires standard analogue amplitude demodulation techniques, which are well known and well established, for example, in radio systems. The proposed observation system can work at a low measurement speed with high accuracy. This reduces the system complexity and timing restraints.

The influence of the shape and size of actuator and sensor is currently unknown and will be a subject of further investigation. An additional benefit of using constant actuation is the ability to use resonant circuits to generate high outputs with low power input. This will be a subject for further investigation, as well.

## Figures and Tables

**Table 1:** Number of irrelevant, partially- or fully-relevant papers, as well as papers that display ultrasonic cure monitoring, but with a pulsed ultrasonic approach. The first 500 search results are sorted by relevance to the search terms “ultrasonic/ultrasound”, “cure”, “monitoring” and “resonant”.

Irrelevant	Pulsed Ultrasonic	Fully/Partially Relevant	Inaccessible
464	32	2	1

**Table 2:** Number of irrelevant, partially- or fully-relevant papers, as well as papers that display ultrasonic cure monitoring, but with a pulsed ultrasonic approach, with respect to the search terms “ultrasound spectroscopy” and “cure monitoring”.

Irrelevant	Pulsed Ultrasonic	Fully/Partially Relevant	Inaccessible
30	0	1	0

## References

[1] Garden L.H., Hayward D., Pethrick R.A. (2007). Dielectric non-destructive testing approach to cure monitoring of adhesives and composites. Proc. Inst. Mech. Eng. Part G.

[2] Maistros G.M., Partridge I.K. (1995). Dielectric monitoring of cure in a commercial carbon-fibre composite. Compos. Sci. Technol..

[3] Chen J.Y., Hoa S.V., Jen C.K., Wang H. (1999). Fiber-Optic and Ultrasonic Measurements for In-Situ Cure Monitoring of Graphite/Epoxy Composites. J. Compos. Mater..

[4] Buggy S.J., Chehura E., James S.W., Tatam R.P. (2007). Optical fibre grating refractometers for resin cure monitoring. J. Opt. A.

[5] Liebers N., Raddatz F., Schadow F. Effective and flexible ultrasound sensors for cure monitoring for industrial composite production. Proceedings of the Deutscher Luft- und Raumfahrtkongress 2012.

[6] Whitney T.M., Green R.E. (1996). Cure monitoring of carbon epoxy composites: An application of resonant ultrasound spectroscopy. Ultrasonics.

[7] Karkanas P. (1998). Cure Modelling and Monitoring of Epoxy/Amine Resin Systems. Ph.D. Thesis.

